# Epidemiological characteristics and antimicrobial resistance of extensively drug-resistant *Acinetobacter baumannii* in ICU wards

**DOI:** 10.1128/spectrum.02619-24

**Published:** 2025-03-04

**Authors:** Jingchao Shi, Xiaoting Mao, Fengtian Sun, Jianghao Cheng, Lijia Shao, Xiaoyun Shan, Yijun Zhu

**Affiliations:** 1 Department of Clinical Laboratory, Affiliated Jinhua Hospital, Zhejiang University School of Medicine (Jinhua Municipal Central Hospital), Jinhua, Zhejiang, China; 2 Department of Clinical Laboratory, Jinhua Maternal and Child Health Care Hospital, Jinhua, Zhejiang, China; 3 Department of Clinical Laboratory, Zhejiang Cancer Hospital, Hangzhou, Zhejiang, China; Icahn School of Medicine at Mount Sinai, New York, New York, USA

**Keywords:** extensively drug-resistant *Acinetobacter baumannii*, PFGE, WGS, ST-208, epidemiological characteristics

## Abstract

*Acinetobacter baumannii* is a significant nosocomial pathogen, particularly problematic due to its extensive drug resistance. This study investigates 56 extensively drug-resistant *A. baumannii* (XDRAB) strains collected from various ICU wards at Jinhua Central Hospital, Zhejiang Province, China. Strains were isolated from diverse clinical samples, including sputum, blood, cerebrospinal fluid, and wound secretions. Identification was confirmed via matrix-assisted laser desorption/ionization-time of flight mass spectrometry (MALDI-TOF MS), and antibiotic susceptibility testing was conducted using the VITEK 2 Compact system, E-test, and Kirby-Bauer methods. All strains were susceptible to polymyxin, with four showing intermediate susceptibility to tigecycline, while resistance rates to other antibiotics were 100%. Molecular typing through pulsed-field gel electrophoresis (PFGE) classified the strains into 10 types, with the dominant type (G) primarily found in ICU3, indicating a potential clonal outbreak. Whole-genome sequencing (WGS) and multi-locus sequence typing (MLST) identified ST208 as the predominant sequence type. Resistance gene screening revealed the presence of blaOXA-23, blaTEM-1D, and aminoglycoside resistance genes in most strains. Phylogenetic analysis confirmed the clonal transmission of ST208 strains across the hospital, with a high degree of genomic similarity among the isolates. These findings highlight the importance of continuous monitoring and effective infection control measures to prevent the spread of XDRAB in healthcare settings.

**IMPORTANCE:**

Extensively drug-resistant *Acinetobacter baumannii* is a critical public health threat, particularly in hospital environments where it causes a variety of infections. The global spread of extensively drug-resistant *A. baumannii* (XDRAB) and its resistance to most antibiotics make treatment options limited, increasing the risk of patient morbidity and mortality. This study provides important insights into the molecular epidemiology of XDRAB in a hospital setting, revealing the clonal transmission of the ST208 sequence type. By utilizing both pulsed-field gel electrophoresis (PFGE) and whole-genome sequencing (WGS), we identified genetic links between strains and the presence of key resistance genes. The findings underscore the urgent need for robust infection control protocols, routine surveillance, and judicious use of antibiotics to mitigate the spread of XDRAB and ensure better patient outcomes.

## INTRODUCTION

*Acinetobacter baumannii* (Ab) is a Gram-negative bacillus that is widely distributed in nature and can colonize various parts of the human body. It has become a significant pathogen responsible for hospital-acquired infections due to its strong adherence properties and ability to survive in harsh environments, making it resistant to conventional disinfection methods. This pathogen is particularly dangerous for immunocompromised patients, leading to a range of infections (1). Due to its ability to evade the action of antimicrobial drugs, the World Health Organization has identified *A. baumannii* as one of the most threatening ESKAPE pathogens, which also includes *Enterococcus faecalis*, *Staphylococcus aureus*, *Klebsiella pneumoniae*, *Pseudomonas aeruginosa*, and *Enterobacteriaceae* (2).

The problem of antibiotic resistance has been exacerbated by the emergence of extensively drug-resistant *A. baumannii* (XDRAB), which shows resistance to a wide array of antibiotics, including the latest generation of antimicrobials. This resistance, combined with its persistence in hospital environments, heightens the risk of nosocomial infections (3). The diversity of resistance mechanisms in *A. baumannii*, along with the presence of mobile genetic elements, facilitates the rapid dissemination of resistance genes among different strains (4).

To address this challenge, bacterial molecular typing technologies play a crucial role in tracking the spread of infectious diseases. By genotyping and analyzing bacterial strains, researchers can trace infection sources and transmission routes, which aids in monitoring prevalence trends and informs the development of preventive measures.

Pulsed-field gel electrophoresis (PFGE) is a high-resolution molecular typing technique that separates large DNA fragments after restriction enzyme digestion, allowing for the distinction between different strains. PFGE is widely used in hospital infection tracking and epidemiological studies due to its ability to reveal subtle genetic differences (5). It is especially valuable in short-term nosocomial outbreak investigations by identifying infection sources and transmission pathways. In contrast, whole-genome sequencing (WGS) offers a broader level of analysis, providing detailed sequence information for the entire bacterial genome. WGS not only identifies resistance genes and mobile elements but also reveals genetic relationships between strains, offering deeper insights into transmission patterns and resistance mechanisms (6). Compared to PFGE, WGS provides more comprehensive data that supports long-term, large-scale epidemiological studies, especially for analyzing the global spread of strains and the evolution of drug resistance.

This study utilizes the strengths of both PFGE and WGS to comprehensively analyze the genetic characteristics and resistance mechanisms of XDRAB. While PFGE is highly effective for tracking hospital outbreaks, WGS contributes to understanding the broader evolutionary and transregional transmission patterns of XDRAB. The combined use of these technologies offers a robust scientific foundation for developing effective control measures and treatment strategies.

## RESULTS

### Overview of the outbreak investigation

This study investigated a hospital outbreak of XDRAB across multiple ICU wards, including ICU Unit 1, ICU Unit 2, ICU Unit 3, Neuro ICU, and EICU. A total of 56 XDRAB strains were isolated from various clinical samples, including sputum, blood, pleural effusion, wound exudates, urine, cerebrospinal fluid (CSF), and bronchoalveolar lavage. The strains were primarily identified as ST208 (Oxford), with additional sequence types (STs) including ST195, ST136, ST469, ST938, and ST369.

XDRAB-ST208 was the predominant strain found across all ICU units, particularly in ICU Unit 3, where the majority of strains were isolated from sputum samples. Genetic analysis using core-genome multi-locus sequence typing (cgMLST) revealed that the XDRAB strains clustered into several groups, with ST208 strains showing high genetic homology and minimal variation, indicating a clonal spread within the hospital. Notably, ST469 was found exclusively in the Neuro ICU, while other STs such as ST195 and ST136 were also present in different units, indicating the spread of multiple clones in various ICU settings.

Resistance gene analysis demonstrated that all 56 strains were resistant to a broad range of antibiotics, including β-lactams, aminoglycosides, fluoroquinolones, and macrolides, with a significant number of strains showing resistance to carbapenems, tetracyclines, and tigecycline. The outbreak of these resistant strains is of particular concern for infection control, as they have been associated with increased morbidity and mortality in ICU settings.

### Strain identification and drug sensitivity results

From January to December 2023, we collected 56 non-repetitive, XDRAB strains from various ICU wards at Jinhua City Central Hospital, Zhejiang Province, China. The distribution of strains by ward was as follows: 4 from the EICU, 8 from ICU1, 5 from ICU2, 24 from ICU3, and 15 from the NSICU. The specimens included 44 from sputum, 2 from lavage fluid, 1 from pleural fluid, 1 from cerebrospinal fluid, 3 from blood cultures, 3 from wound secretions, and 2 from urine. All 56 strains were identified as *A. baumannii* using matrix-assisted laser desorption/ionization-time of flight mass spectrometry (MALDI-TOF MS), with identification scores exceeding 2.0. Drug susceptibility testing was performed using the VITEK 2 Compact system with the GN335 card, and results were validated using E-test and Kirby-Bauer methods, according to Clinical and Laboratory Standards Institute (CLSI) guidelines (FDA standards were used for tigecycline). All 56 XDRAB strains were susceptible to polymyxin (100%), while 52 strains (93%) were susceptible to tigecycline, and four strains (7%) showed intermediate susceptibility to tigecycline. These four isolates (AB6, AB28, AB42, and AB63) were identified as ST208. Resistance rates to all other tested antibiotics, including piperacillin/tazobactam, cefoperazone/sulbactam, Ticarcillin/clavulanic, ceftazidime, Cefepime, Imipenem, meropenem, Tobramycin, levofloxacin, Ciprofloxacin, Cotrimoxazole and levofloxacin, were 100%. This uniform resistance profile highlights the extensive drug resistance of the isolates.

### Pulsed-field gel electrophoresis results

**Fig 1:**
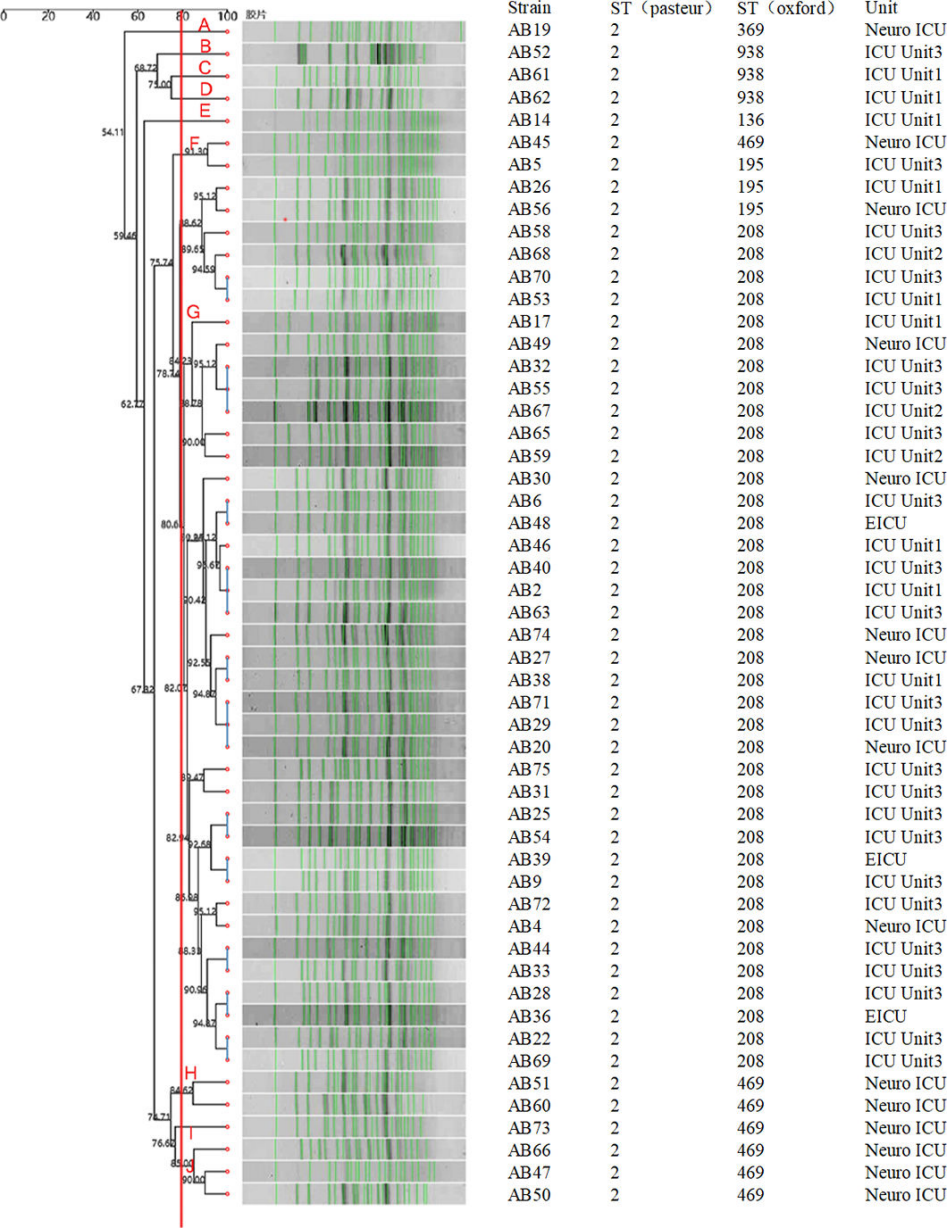
PFGE clustering of 53 pan-resistant *Acinetobacter baumannii* strains. The red line represents the 80% similarity threshold.

PFGE analysis using ApaI digestion for *A. baumannii* and XbaI for Salmonella typhimurium H9812 as the standard strain successfully produced band profiles for 53 of the 56 strains. Despite repeated attempts, three strains exhibited persistent DNA degradation, rendering their PFGE results unavailable. Setting the similarity threshold at 80%, the remaining strains were categorized into 10 PFGE types (A–J). Type G contained the majority (40 strains), followed by type J (three strains), and types F and H (two strains each). Types A, B, C, D, E, and I contained one strain each. The predominance of G-type strains in ICU3 and their presence in other ICUs suggests a potential clonal outbreak. Notably, most G-type strains were of the ST208 sequence type, supporting the hypothesis of clonal transmission across the hospital. Other PFGE types (F, H, A, B, C, D, E, I) were distributed across various wards, possibly indicating independent origins or cross-infections between patients. This suggests that a clonal outbreak, predominantly involving ST208 strains, occurred in ICU3, likely linked to healthcare practices or environmental factors (Fig. 1 for PFGE clustering).

### Average nucleotide identity and MLST typing results

**Fig 2:**
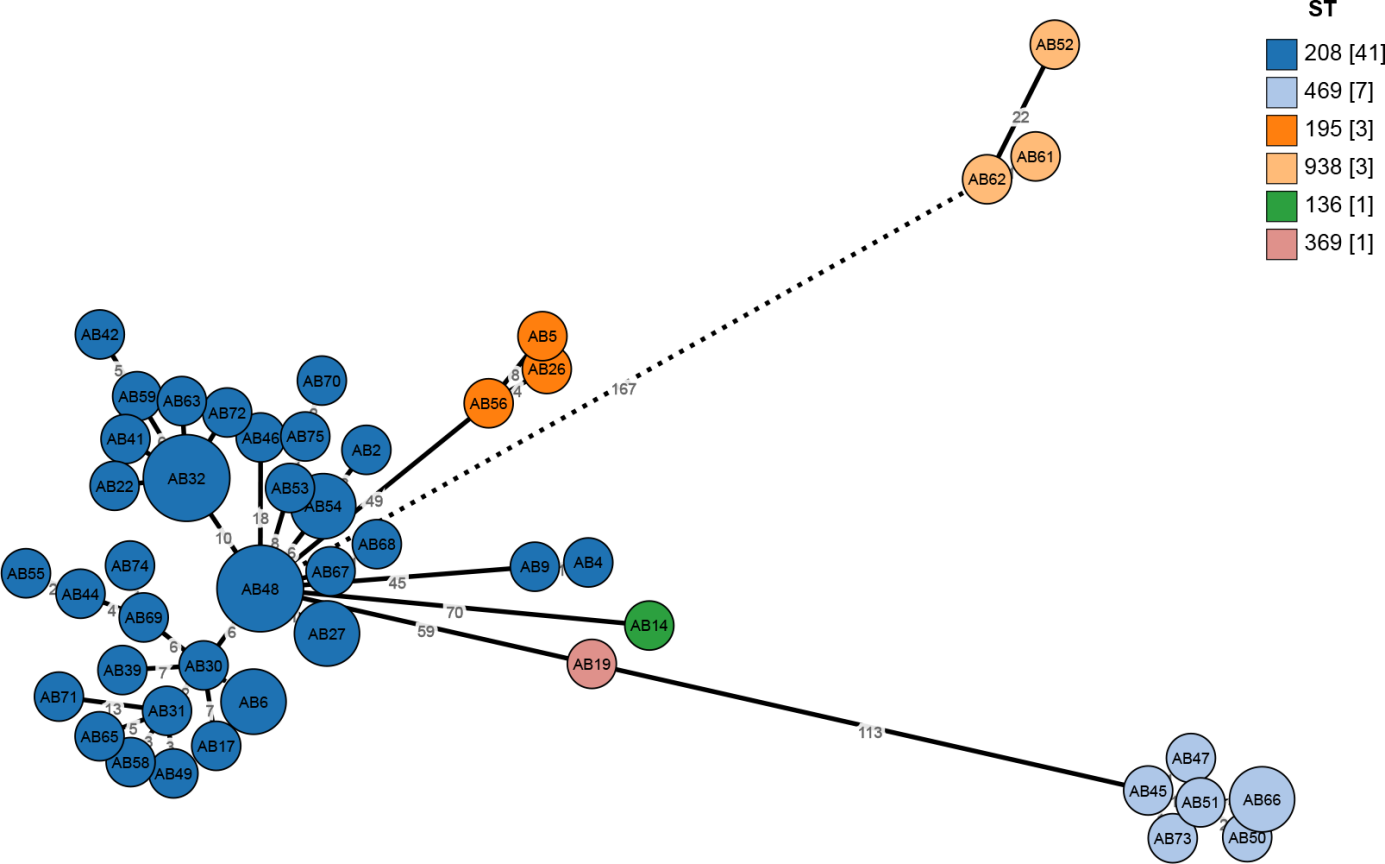
Minimum spanning tree of 56 XDRAB strains. The tree was constructed using cgMLST, with the distance between each node representing the clonal relationship between species. Different STs are indicated by colored circles. Numbers in square brackets represent the number of isolates within each ST.

Average nucleotide identity analysis revealed that the genomic similarity among all 56 XDRAB strains exceeded 99.8%, confirming their high genomic concordance and classification as *A. baumannii*. Multi-locus sequence typing (MLST) typing using the PUBMLST website showed that all strains belonged to ST2 according to Pasteur typing, while Oxford typing differentiated them into multiple sequence types: ST136, ST195, ST208, ST369, ST469, and ST938. Oxford typing was used for further analysis due to its superior resolution. ST208 was the dominant type (41 strains, 73%), followed by ST469 (seven strains), ST195 and ST938 (three strains each), and ST136 and ST369 (one strain each). eBURST analysis indicated that all strains belonged to the clonal complex CC208 (also known as CC92). cgMLST typing revealed a broader range of types, with strains categorized into nine types, including 1325, 1630, 2254, 2171, and 4801, from which a minimum spanning tree was constructed (Fig. 2).

### Phylogenetic analysis and comparative resistance gene analysis

We performed a phylogenetic analysis of the 56 XDRAB strains based on single nucleotide polymorphisms (SNPs) in the coding regions, using *Acinetobacter baumannii* ATCC17978 as the reference genome. The phylogenetic tree revealed three distinct clusters. Clusters 2 and 3 consisted primarily of Oxford ST208 strains, which showed high genetic homology and minimal variation. In contrast, cluster 1 included multiple STs, such as ST938, ST469, ST136, ST369, ST208, and ST195. Notably, ST469 was found exclusively in the neurosurgical ICU, whereas ST208, the predominant strain in the hospital, was primarily located in ICU3 and was also detected in other ICU wards.

Resistance gene analysis using the ResFinder and CARD databases showed that all 56 strains harbored β-lactamase-related genes, including the class D blaOXA-23 (acquired), blaOXA-66 (intrinsic), and the class C blaADC-25 (intrinsic). blaOXA-23, a key determinant of resistance to carbapenem antibiotics, explains the observed 100% resistance to meropenem and imipenem in all strains. Additionally, blaOXA-66 and blaADC-25, both intrinsic to *A. baumannii*, contribute to partial resistance to cephalosporins and piperacillin/tazobactam, though their effects are weaker than OXA-23.

Moreover, 48 strains (86%) carried the class A β-lactamase gene blaTEM-1D (acquired), which mediates resistance to β-lactam antibiotics, including cephalosporins and piperacillin/tazobactam, further contributing to resistance.

All strains also contained aminoglycoside resistance genes such as ANT(3'')-IIc (intrinsic), aph(3'')-Ib (intrinsic), and aph(6)-Id (intrinsic), with 43 strains (77%) carrying aph(3')-Ia (acquired). These genes explain the 100% resistance to aminoglycosides, such as tobramycin and gentamicin. The 16S rRNA methyltransferase gene armA (acquired), conferring high-level resistance to aminoglycosides, was present in all strains, further contributing to aminoglycoside resistance.

Mutations in gyrA (S81L) and parC (S84L, V104I, D105E) were detected in all 56 strains, conferring resistance to fluoroquinolones (e.g., levofloxacin and ciprofloxacin). These mutations explain the 100% resistance to fluoroquinolones observed in our study.

Moreover, all strains harbored macrolide resistance genes mph(E) (intrinsic) and msr(E) (intrinsic), which encode methylase and efflux proteins, respectively. These genes contribute to 100% resistance to macrolides (e.g., erythromycin and azithromycin).

Tetracycline resistance was mediated by tet(B) (intrinsic) and its regulatory gene tetR (intrinsic) in all strains. This mechanism was activated upon tetracycline exposure, contributing to the observed resistance.

The 7% of isolates with intermediate susceptibility to tigecycline (e.g., AB6, AB28, AB42, AB63) showed alterations in efflux pump activity or target modifications, which may contribute to reduced tigecycline susceptibility. The precise mechanism of this intermediate resistance remains to be fully elucidated but is likely related to variations in efflux pump regulation or target binding.

**Fig 3:**
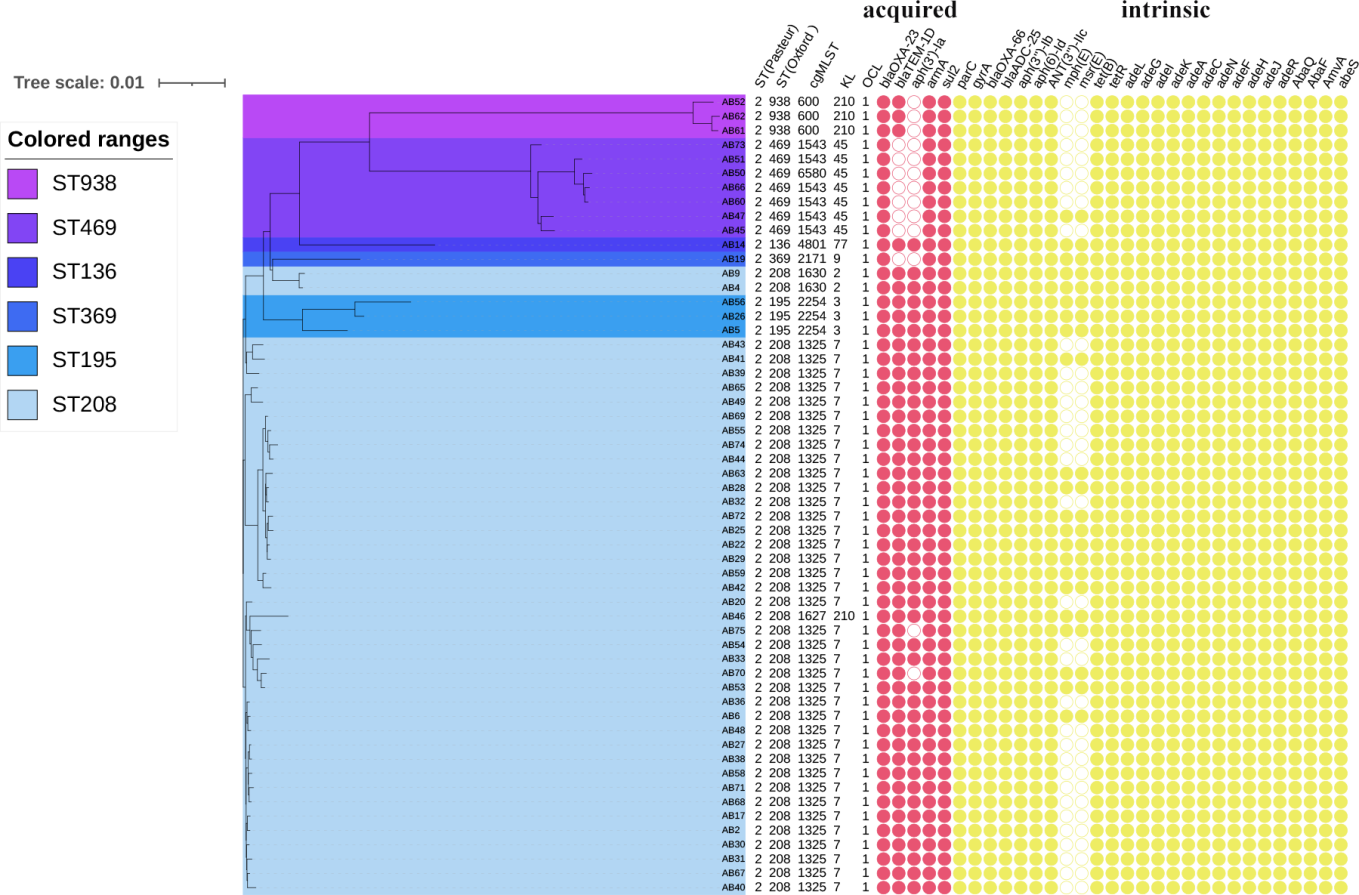
Phylogenetic tree of 56 XDRAB strains based on SNP analysis. The phylogenetic relationships between isolates were determined through SNP analysis. Different color thresholds were used to mark XDRAB strains with distinct ST types. Additionally, the presence or absence of resistance genes is shown using filled and unfilled circles, respectively. Acquired resistance genes are represented in red, while intrinsic resistance genes are shown in yellow. The figure also includes capsular polysaccharide loci (KL) and lipo-oligosaccharide outer core synthesis loci (OCL) typing information for the isolates.

Various efflux pump genes were also detected, including those from the AdeABC, AdeIJK, and AdeFGH families of the Resistance-Nodulation-Division superfamily, as well as pumps from the Small Multidrug Resistance and Major Facilitator superfamilies. These efflux pumps potentially contribute to resistance against β-lactams, macrolides, fluoroquinolones, tetracyclines, and certain disinfectants, such as chlorhexidine and benzalkonium chloride (see Fig. 3 for the phylogenetic tree of resistance genes, with intrinsic and acquired genes color-coded).

### Virulence gene and mobile element screening

Using the VFDB database and Kaptive, we identified the capsular polysaccharide loci (KL) and lipo-oligosaccharide outer core synthesis loci (OCL) in the genome sequences of the 56 XDRAB strains. All strains contained virulence genes related to multiple mechanisms, with only two strains lacking the cusA and cusA/B genes. Additionally, all strains possessed the katA gene, commonly found in Neisseria spp., which aids in survival under oxidative stress by protecting against reactive oxygen species. Among the podoplanar polysaccharides, KL7 was the most prevalent type (38 strains), with other types including KL2, KL3, KL9, KL45, KL77, and KL210. All strains harbored the OCL1 type of lipo-oligosaccharide outer core.

**Fig 4:**
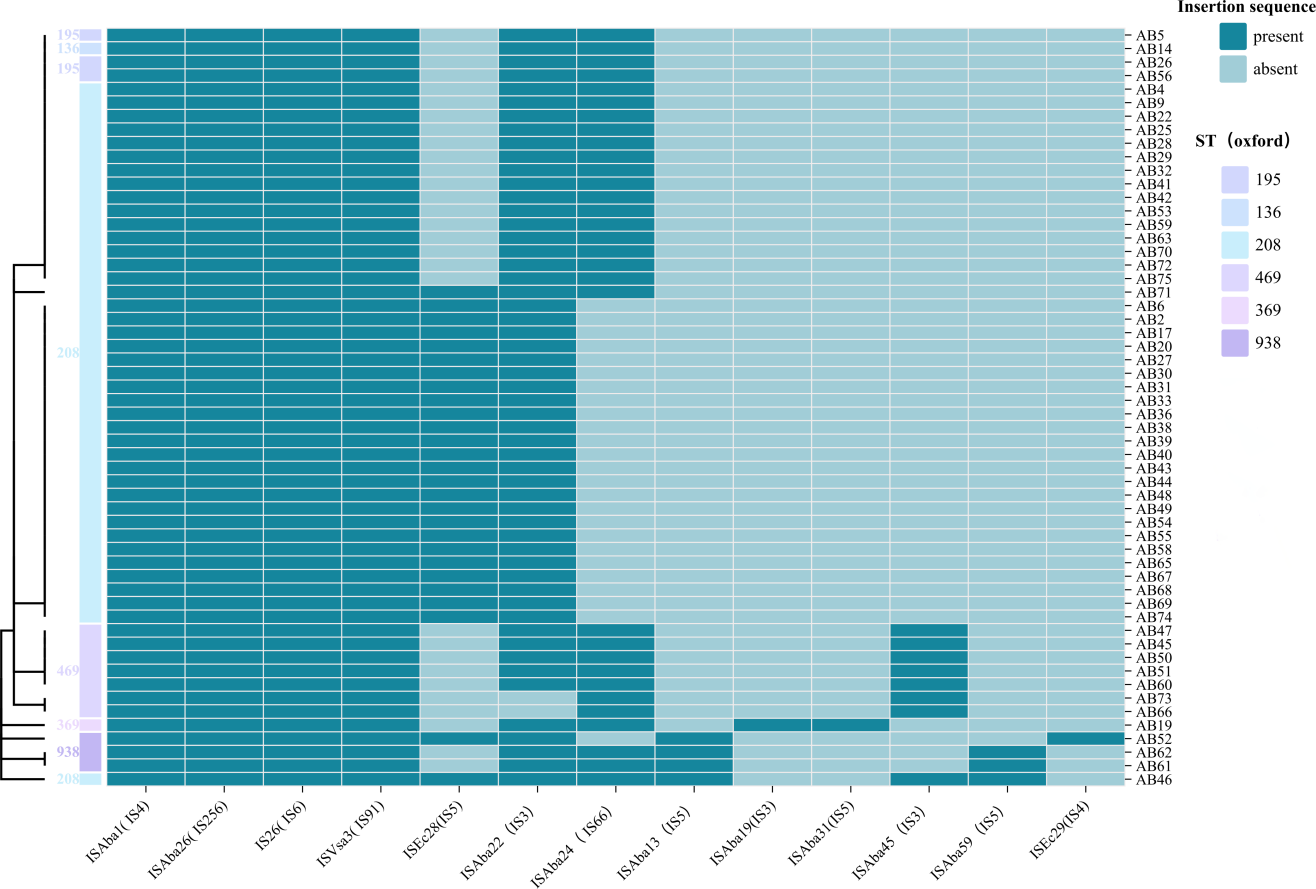
Genetic heatmap of insertion sequences in 56 XDRAB strains. Darker colors represent the presence of insertion sequences, while lighter colors indicate absence. The ST types are displayed on the left, and clustering analysis was performed to group similar strains.

Mobile element analysis using MobileElementFinder, ISfinder, INTEGRALL, and BacAnt tools (with identity and coverage set above 90%) revealed no integrons in any strain. Thirteen insertion sequences were identified, with ISAba1, ISAba26, IS26, and ISVsa3 found in all strains, while ISAba22 was present in 54 strains. Additionally, ISAba24 was found in 31 strains, ISEc28 in 27, ISAba45 in 8, ISAba13 in 4, and ISAba19 and ISAba31 in 1 strain each. We also detected transposons such as Tn6022 and Tn2006. Two non-conjugative plasmids, ABkp1 (rP-T1 type) and pAB0057 (r3-T1 type) were identified across all strains, as indicated by the absence of the origin of transfer (oriT) site (Fig. 4).

### Geographic dissemination of ST208

**Fig 5:**
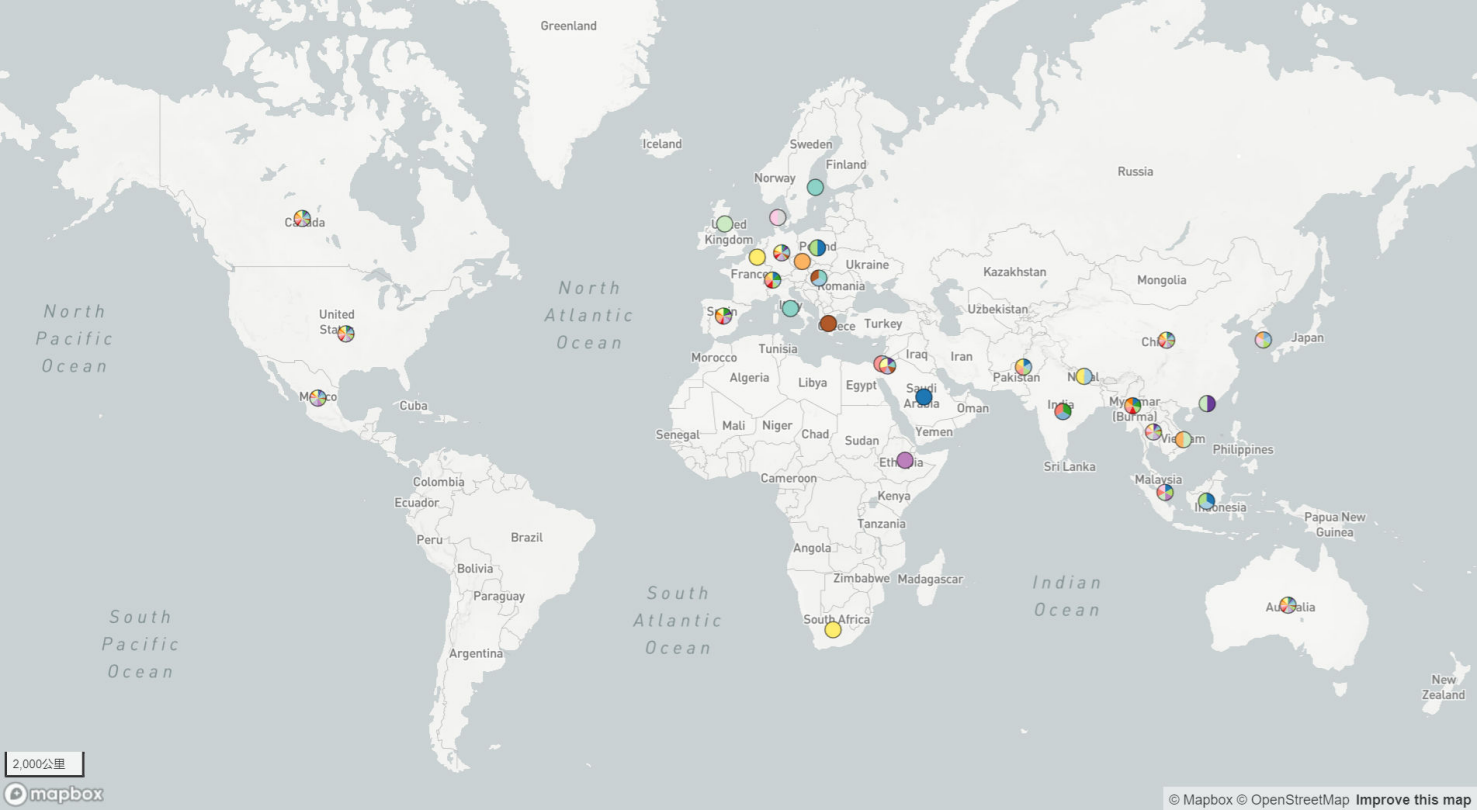
Global distribution of *A. baumannii* ST208 types. Visual representation provided by microreact.org.

**Fig 6:**
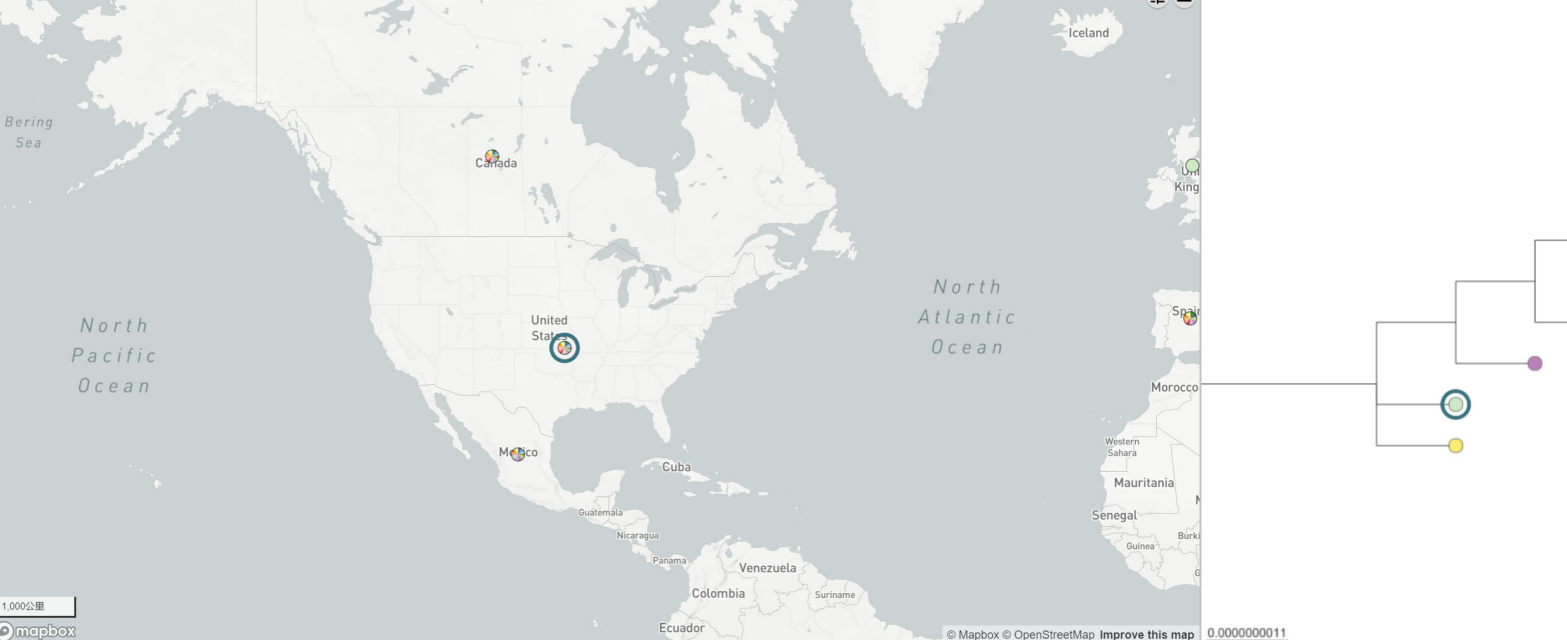
Phylogenetic analysis suggests the possible origin of ST208 in North America. Visual representation provided by microreact.org.

**Fig 7:**
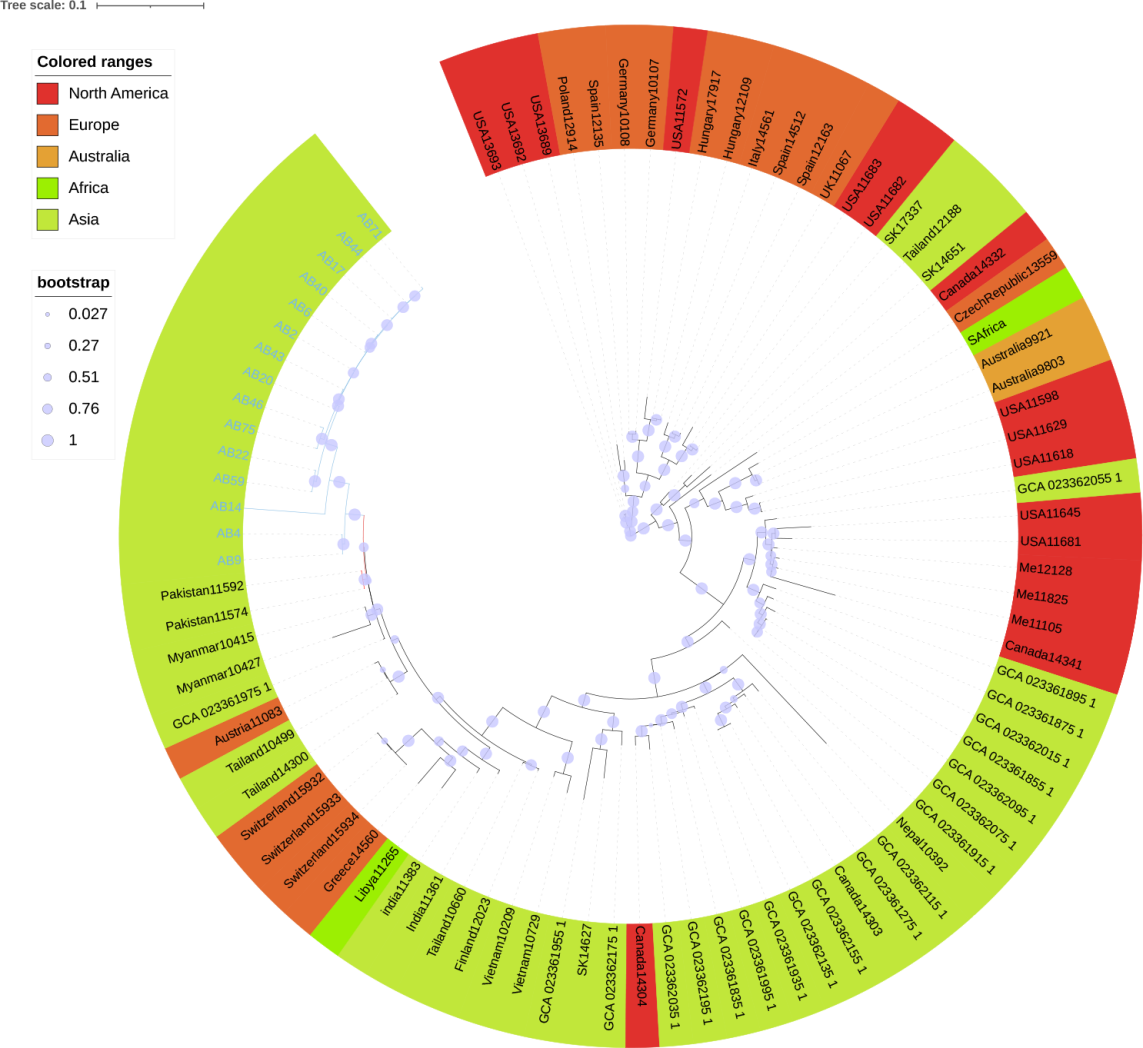
Phylogenetic tree of 90 ST208 strains. The phylogenetic tree highlights ST208 strains from different continents, each color-coded accordingly. The 15 strains included in this study are marked in blue. The bootstrap values indicate the stability of the phylogenetic tree.

We conducted a geographic dissemination analysis of the AB-ST208 strain, the predominant cause of clonal outbreaks in our hospital. Phylogenetic analysis using BIGSdb and visualized with Microreact (7) revealed the global spread of AB-ST208 across five continents: North America, Europe, Asia, Australia, and Africa. The United States and China had the highest reported incidences of AB-ST208 (Fig. 5). The strain was first reported in Singapore in 1996, and phylogenetic evidence suggests that the global spread originated in North America. Minimal evolutionary tree analysis using PubMLST showed that AB-ST208 is the most commonly detected sequence type globally, with Hangzhou, Zhejiang Province, China, having the highest detection rate (Fig. 6). Comparative analysis of 75 AB-ST208 strains from NCBI and PubMLST, along with 15 strains from this study, was conducted to explore the genetic relationships between the isolates. The additional genomes were selected based on geographic diversity, genetic homology, and data quality. The analysis revealed that the strain Pakistan11592 from Pakistan was the closest related strain to the XDRAB-ST208 strains from this study (Fig. 7).

## DISCUSSION

The origin and distribution of XDRAB strains are crucial for understanding the transmission and infection mechanisms of this pathogen. In this study, we collected 56 XDRAB strains from various clinical specimens across multiple ICU wards in the hospital, including sputum, lavage fluid, pleural fluid, cerebrospinal fluid, blood, wound secretions, and urine. This broad range of sample sources highlights the potential transmission of XDRAB at different infection sites and suggests that transmission may be influenced by factors such as patient health status, hospital environment, and medical practices. Antibiotic susceptibility testing revealed that XDRAB demonstrated 100% resistance to all antibiotics except polymyxin and tigecycline, posing a significant challenge to clinical management and limiting treatment options, which may result in treatment failure and worsened patient outcomes.

To assess the relationships between strains from different origins and minimize potential clonal outbreaks, it is essential to use effective strain typing methods. Several methods exist for typing *A. baumannii*, including PFGE, multiple-locus variable-number tandem-repeat analysis, amplified fragment length polymorphism, and MLST. Among these, PFGE has been recognized internationally as the gold standard for investigating nosocomial outbreak strains, owing to its high resolution (8). PFGE distinguishes DNA fragments ranging from 30 kb to 1 Mb in size, typically displaying 20 to 25 bands. By comparing the similarity of banding patterns, the relatedness of strains can be inferred: strains with near-identical PFGE profiles are usually epidemiologically linked and may originate from the same clone. In contrast, strains showing distinct banding patterns are considered less related. In this study, PFGE analysis using the ApaI enzyme classified 53 XDRAB strains into 10 distinct PFGE types, with type G being the most prevalent, particularly in ICU3. This finding suggests a clonal outbreak in ICU3 that later spread to other ICU wards.

While PFGE is valuable for short-term epidemiological investigations, it is limited by the natural evolution of strains over time. Genetic changes, such as gene locus modifications, may occur under external pressures, reducing the method’s effectiveness in long-term studies. Additionally, PFGE is labor-intensive, time-consuming, and subject to interpretive variability, limiting its inter-laboratory comparability. In contrast, MLST, which sequences multiple housekeeping genes, offers a more standardized and widely applicable approach for data comparison across regions and laboratories. Although MLST lacks the ability to detect minor differences as effectively as PFGE, its reproducibility and generalizability make it a preferred method in many cases.

In our study, we observed that all XDRAB strains classified as PFGE type G were also identified as ST208 by MLST. However, PFGE type F strains included multiple sequence types, such as ST469, ST195, and ST208, indicating discrepancies between PFGE and MLST results. These inconsistencies may stem from differences in resolution, genomic structural changes, horizontal gene transfer, recombination events, or technical limitations. Thus, a combination of typing methods is necessary to comprehensively understand the genetic characteristics and transmission dynamics of bacterial strains.

WGS has emerged as a powerful tool for elucidating the genetic characteristics and molecular epidemiology of pathogens. In this study, WGS revealed a high degree of genomic similarity (>99.8%) among the 56 XDRAB strains. MLST analysis identified six major sequence types: ST208, ST136, ST195, ST369, ST469, and ST938. eBURST analysis indicated that all strains belonged to the clonal complex CC208 (also known as CC92), a widespread clonal complex frequently associated with carbapenem resistance genes, such as blaOXA-23-like, blaOXA-40-like, and blaOXA-58-like. CC208 had a significantly higher rate of resistance to carbapenemase antibiotics than the non-CC208 group (9). Consistent with this, all XDRAB strains in our study carried the blaOXA-23 gene. Phylogenetic analysis using cgMLST established a minimum spanning tree, demonstrating that ST208 was the dominant clone, primarily in ICU3, and had disseminated throughout the ICU wards. Although ST469 was only detected in one ICU, it requires close monitoring to prevent further spread.

There is significant genomic variability in AB. To accurately assess the relatedness of XDRAB strains, we conducted a phylogenetic analysis based on SNPs. Cluster 1 contained several STs, including ST938, ST469, ST136, ST369, ST208, and ST195, indicating genetic diversity within this group. In contrast, clusters 2 and 3 consisted solely of ST208 strains, reflecting higher genetic homology and less variation, consistent with their widespread presence in hospitals. Resistance gene profiling revealed that both cluster 2 and cluster 3 strains harbored class A (TEM-1D), class C (ADC-25), and class D (OXA-23, OXA-66) β-lactamases. However, ST369 and ST469 strains in cluster 1 lacked the TEM-1D gene, and ST208, ST195, and ST136 strains lacked aph(3')-Ia and mph(E), msr(E). The mechanism behind the loss of resistance genes in multidrug-resistant bacteria remains unclear but may involve pseudogenization due to deleterious mutations. When resistance genes are no longer necessary for survival, such mutations can inactivate the genes, reducing the energy burden on the bacterium and improving fitness (10).

In this experiment, XDRAB strains exhibited resistance to nearly all antibiotics, except for polymyxin and tigecycline. This resistance pattern aligns with our findings of a high prevalence of resistance genes, including β-lactamase, aminoglycoside, cephalosporinase, sulfonamide, tetracycline, macrolide, and various efflux pump genes. Among these, class D enzymes, also known as oxacillinases (OXA enzymes), represent the primary resistance mechanism in AB. OXA-51, which is naturally present in *A. baumannii*, has a limited ability to hydrolyze carbapenems. In our study, OXA-66, a member of the OXA-51 family, was identified. Interestingly, recent research indicates that specific amino acid substitutions, such as W222G and I129L, can significantly enhance the catalytic activity of OXA-66, resulting in greater efficacy against carbapenem antibiotics than OXA-23 and OXA-24/40 (11). Notably, OXA-23 is widely regarded as the most significant contributor to carbapenem resistance in AB (12). The presence of the insertion sequence ISAba1 enhances OXA-23 expression, and resistance increases with the presence of multiple mobile elements in OXA-23-positive strains (13). Furthermore, OXA-23 can associate with adjacent copy sequences to form a composite transposon, mediating the horizontal transfer of resistance genes. In our study, both OXA-23 and ISAba1 were detected in all XDRAB strains. The OXA-23 gene is found on the chromosome and/or plasmid and has been associated with four genetic constructs: Tn2006, Tn2007, Tn2008, and Tn2009 (14). Among the 56 XDRAB strains in our study, several insertion sequences were identified, with ISAba1, ISAba26, IS26, and ISVsa3 being the most common. The transposition of insertion sequences or transposons contributes to antibiotic resistance by altering bacterial gene expression, such as introducing strong promoter sequences for downstream genes, disrupting promoters to decrease expression, or inactivating genes (15). A study reported that the presence of blaTEM-1D and ISAba1-ampC significantly increased sulbactam resistance in AB isolates, and resistance was further elevated in strains carrying the IS26-blaTEM-1D-Tn3-IS26 gene assembly (16). This correlates with the high resistance to cefoperazone/sulbactam observed in the XDRAB strains in our study.

In our study, nearly all XDRAB strains contained the core virulence genes of *A. baumannii*, with the KatA gene present in all strains. The KatA gene enables *A. baumannii* to withstand oxidative stress from various environments, facilitating immune evasion. Additionally, capsular polysaccharides play a crucial role in enhancing resistance to disinfection and prolonged desiccation, aiding *A. baumannii*’s survival in hospital environments. Both K-antigen (capsular polysaccharide) and O-antigen surface polysaccharides are key virulence factors, protecting *A. baumannii* from complement-mediated phagocytosis. Notably, significant variations in the composition and structure of K antigens may occur among different strains of the same species (17, 18). Increased transcription of K genes leads to higher production of these antigens; however, due to their diversity, the clinical significance of variations at different K loci remains unclear. Currently, KL2 is the most prevalent *A. baumannii* strain type globally, whereas KL7 is predominantly found in China. This is consistent with the high proportion of KL7 observed in our study.

According to PUBMLST, ST208 *A. baumannii* was first reported in Singapore in 1996, after which it spread to Australia, the United States, Europe, and various Asian countries. It has since become one of the predominant STs of carbapenem-resistant isolates in China (19). Global data analysis indicates that ST208 is now the most common ST of *A. baumannii*, with North America likely being its origin. The United States and China report the highest detection rates, consistent with previous studies on ST208 (20). Further analysis at the city level revealed that Hangzhou, China, had the highest detection rate of ST208, which may be related to factors such as the number of healthcare facilities, patient volume, and antibiotic usage. In our study, ST208 was also the most frequently detected type, particularly due to the geographic proximity and mobility between our region and Hangzhou. This suggests that the epidemiological trends of ST208 in Hangzhou may reflect patterns throughout Zhejiang Province. Additionally, our phylogenetic analysis of 75 ST208 strains globally and 15 XDRAB-ST208 strains from our study demonstrated that ST208 disseminated from North America to Europe, Australia, and Asia. Notably, the XDRAB-ST208 strains in our study were phylogenetically closest to strain Pakistan11592 from Pakistan, potentially reflecting the role of globalization, transnational transmission, and patient movement between healthcare systems.

In this study, we conducted an in-depth molecular epidemiological investigation of nosocomial XDRAB. Two key molecular epidemiology tools, PFGE and WGS, played essential roles. PFGE provided genetic “fingerprints” to reveal similarities and differences between strains, identifying G-type XDRAB as a potential source of clonal transmission within our ICU. Meanwhile, WGS offered more comprehensive genomic insights, allowing for a deeper understanding of XDRAB’s molecular characteristics, transmission pathways, and epidemiological dynamics, as well as its resistance and virulence genes. The genetic context of resistance in XDRAB was analyzed using short-read sequencing technologies, specifically Illumina sequencing. While short-read sequencing offers high accuracy and enables comprehensive genomic analysis, it has certain limitations in resolving complex genomic structures, such as plasmids and mobile genetic elements (MGEs). Short reads are often unable to effectively assemble regions with high GC content, repetitive sequences, or those involving large structural variations, such as plasmids or integrons. As a result, the identification of plasmid-associated genes and mobile elements is often incomplete or fragmented. To address these limitations, we employed computational tools like PlasmidFinder and the CARD database to predict plasmid-associated genes and resistance elements from the short-read data. While this approach provided useful insights, it is not as comprehensive as the analysis enabled by long-read sequencing technologies (e.g., PacBio or Oxford Nanopore). Long-read sequencing has the advantage of providing longer contiguous sequences, allowing for more accurate assembly of plasmids and better resolution of mobile genetic elements that are difficult to capture with short reads. This would improve the understanding of horizontal gene transfer and the role of plasmids in the spread of resistance, as well as provide a clearer view of the plasmid structures and MGEs present in the strains. Although short-read sequencing allowed us to perform high-throughput and relatively cost-effective analysis of the genetic content in XDRAB, the full characterization of plasmids and MGEs will require long-read sequencing in future studies. By incorporating long-read sequencing, future research could provide a more complete understanding of the genetic context, the role of plasmids in resistance dissemination, and the dynamics of mobile elements in the evolution and spread of XDRAB.

Effective nosocomial infection control involves regular pathogen surveillance, especially for multidrug-resistant strains, and strict adherence to infection control protocols like hand hygiene and patient isolation. Healthcare workers must receive continuous training to follow proper procedures, reducing the risk of transmission. Optimizing the use of antimicrobial drugs through strict guidelines is critical to preventing resistance. Additionally, enhanced patient monitoring, particularly for those with resistant strains like ST208 *A. baumannii*, and maintaining infection control records will help improve future prevention strategies.

## MATERIALS AND METHODS

### Strain origin and identification

Fifty-six non-repetitive, XDRAB strains were selected from various clinical specimens collected across multiple ICU wards at Jinhua Central Hospital, Zhejiang Province, China, between January and December 2023. The selection criteria for clinical isolates were as follows:

#### Inclusion criteria

Patient population: only strains isolated from patients diagnosed with severe hospital-acquired infections were included. This included patients with respiratory tract infections, bloodstream infections, wound infections, and urinary tract infections who were admitted to different ICU wards.

XDR definition: the isolates were required to meet the definition of XDR, showing resistance to at least one agent from three or more antibiotic classes, including β-lactams, aminoglycosides, fluoroquinolones, carbapenems, and macrolides.

Sample type: clinical specimens included sputum, blood, pleural fluid, CSF, urine, and wound secretions to represent diverse infection sites.

Sample quality: only high-quality, non-repetitive isolates were included to ensure that each strain was genetically distinct and represented a unique infection event.

#### Exclusion criteria

Non-*A*. *baumannii* strains: strains belonging to other groups within the Acinetobacter complex were excluded.

Inadequate clinical data: strains from patients with insufficient clinical data (e.g., missing infection site or patient history) were excluded to ensure the clarity and reliability of the findings.

##### Identification and confirmation

Strain identification was confirmed using MALDI-TOF MS, with a score of >2.0 to ensure accurate species identification. Quality control strains included *Escherichia coli* ATCC 25922, *Pseudomonas aeruginosa* ATCC 27853, and *Staphylococcus aureus* ATCC 25923 to ensure proper identification and testing procedures.

### Antibiotic susceptibility testing

Antibiotic susceptibility was assessed using the VITEK 2 Compact system. Bacterial suspensions were adjusted to a 0.5 McFarland standard, diluted accordingly, and loaded onto GN-335 cards. Minimum inhibitory concentrations (MICs) were automatically read after 16–20 hours. MICs for imipenem, meropenem, and levofloxacin were confirmed using E-test methods, while tigecycline and polymyxin were tested using microbroth dilution. Questionable VITEK 2 results were verified by the Kirby-Bauer method. Susceptibility results were categorized as sensitive (S), intermediate (I), or resistant (R) following CLSI guidelines (21). XDRAB isolates were stored on Mueller-Hinton agar plates and preserved in 30% glycerol at −80°C for further testing.

### Pulsed-field gel electrophoresis

Single colonies from XDRAB isolates stored at −80°C were cultured and adjusted to a concentration of 4.0 McFarland. Samples were treated with Proteinase K and incorporated into a 1% Seakem Gold agarose gel. The gel blocks underwent enzymatic digestion and were electrophoresed for 18 hours in TBE buffer. Post-electrophoresis, the gel was stained with GelRed, decolorized in water, and imaged. Clustering analysis was performed using BioNumerics software, with a similarity threshold of ≥80% indicating identical clones (22).

### Bacterial genomic DNA extraction

Genomic DNA was extracted from XDRAB isolates using the MiniBEST Bacterial Genomic DNA Extraction Kit. Single colonies were transferred to LB broth for enrichment, and following centrifugation, cells were lysed in SP buffer with RNase A. Lysozyme and EDTA were added for lysis, and DNA was precipitated after adding Solution A and Solution B. The DNA was purified using a filter cup and spin column, washed, eluted in distilled water, and stored at −20°C.

### Whole-genome sequencing

Whole-genome sequencing was performed on DNA from the 56 XDRAB isolates using the Illumina sequencing platform with 2 × 150 bp paired-end reads. A total of 0.2 µg DNA per sample was fragmented to a size of 350 bp, end-repaired, A-tailed, adapter-ligated, and PCR-amplified. Library quality was assessed using the Agilent 5400 system, and the qualified libraries were pooled and sequenced. The sequencing was conducted using the Illumina NovaSeq 6000 platform, ensuring high-quality data with an average coverage depth of ~100×. Fastp (version 0.23.1) was used to process the raw data, performing quality control by removing low-quality reads, adapter contamination, and reads with >10% uncertain nucleotides.

### Sequencing data analysis

Genome assembly was performed using SPAdes software (23), and annotation was completed using the RASTtk tool (24). MLST and cgMLST were conducted via the PUBMLST platform (25) (https://pubmlst.org/organisms/acinetobacter-baumannii), with cgMLST providing higher resolution. Resistance genes were identified using Resfinder (26) (http://genepi.food.dtu.dk/resfinder) and the CARD RGI tool (27) (https://card.mcmaster.ca/analyze/rgi), with similarity and length thresholds set at 90%. Virulence genes were detected with VFanalyzer (28) (http://www.mgc.ac.cn/VFs), and Kaptive (29) was used to analyze capsular polysaccharide and lipooligosaccharide loci. Mobile elements were identified using MobileElementFinder (30), with rep genes used to detect plasmids. Phylogenetic relationships were analyzed using BacWGSTdb’s Clearcut software (31, 32), and minimum spanning trees were visualized using Grapetree (33).

For the WGS-based SNP analysis, relatedness between strains was defined using SNPs. Strains that differed by fewer than 20 SNPs were considered closely related, indicating a possible clonal origin. In contrast, strains differing by more than 100 SNPs were considered genetically distinct. This approach allowed us to track potential transmission pathways and assess the degree of genetic diversity within the hospital.

## Data Availability

The genome sequencing data are publicly available at NCBI GenBank under the BioProject accession number PRJNA1172120.
